# A human-like model of aniridia-associated keratopathy for mechanistic and therapeutic studies

**DOI:** 10.1172/jci.insight.183965

**Published:** 2024-12-03

**Authors:** Dina Javidjam, Petros Moustardas, Mojdeh Abbasi, Ava Dashti, Yedizza Rautavaara, Neil Lagali

**Affiliations:** Division of Ophthalmology, Department of Biomedical and Clinical Sciences, Linköping University, Linköping, Sweden.

**Keywords:** Ophthalmology, Stem cells, Genetic diseases, Mouse models

## Abstract

Aniridia is a rare congenital condition of abnormal eye development arising principally from heterozygous mutation of the *PAX6* gene. Among the multiple complications arising in the eye, aniridia-associated keratopathy (AAK) is a severe vision-impairing condition of the cornea associated with a progressive limbal stem cell deficiency that lacks suitable treatment options. Current mouse models of aniridia do not accurately represent the onset and progression dynamics of human AAK, hindering therapy development. Here, we performed deep phenotyping of a haploinsufficient *Pax6*^+/–^ small-eye (Sey) mouse model on the 129S1/SvImJ background, which exhibits key features of mild presentation at birth and progressive AAK with aging, mimicking human disease. The model exhibits a slowly progressing AAK phenotype and provides insights into the disease, including disturbed basal epithelial cell organization, function, and marker expression; persistent postnatal lymphangiogenesis; disrupted corneal innervation patterns; and persisting yet altered limbal stem cell marker expression with age. The model recapitulates many of the known features of human disease, enabling investigation of underlying disease mechanisms and, importantly, access to a well-defined temporal window for evaluating future therapeutics.

## Abstract 2

We present a mouse model of a blinding eye disease, recapitulating the human disease course to enable therapy development.

## Introduction

Congenital aniridia is a disorder of disrupted normal eye development owing to haploinsufficiency of the PAX6 protein caused by heterozygous mutations of the *PAX6* gene (1). Underdeveloped eye structures lead to multiple ocular pathologies, including iris and foveal hypoplasia. Additionally, early-onset glaucoma and cataracts are reported, which may arguably be attributed to abnormal trabecular meshwork differentiation (1, 2). Importantly, in most cases, the development of aniridia-associated keratopathy (AAK) becomes the vision-limiting factor. AAK is a slow-onset, progressive loss of corneal transparency associated with inflammation, neovascularization, and an insufficiency of limbal stem cells (LSCs) that normally constantly renew the corneal epithelium. The prevalence and severity of AAK tend to increase as patients age, with progressive LSC deficiency, pervasive inflammation, and neurodegeneration present in the cornea and with surgical interventions unfortunately proving ineffective (3, 4), leading to blindness with poor prognosis for subsequent interventions (5). To develop more effective and targeted therapies, a deeper understanding of the mechanisms leading to AAK and its progression is needed — thus, a relevant and accurate model of AAK is essential.

Mice are an excellent model for congenital aniridia as the human *PAX6* and mouse *Pax6* genes are identical and encode the same amino acid sequence. Transgenic mice with an aniridia phenotype are created by targeted mutation of 1 allele of the *Pax6* gene (called the Sey allele, for “small-eye”), resulting in mutant mice exhibiting a spectrum of AAK phenotypes. As about 70% of all human cases of aniridia result from premature termination codon (PTC) mutations leading to nonsense-mediated PAX6 protein decay (6, 7), this mutation type is the most useful for investigating disease mechanisms. Potential therapeutics also exist for this type of mutation, such as nonsense-suppression drugs (also called read-through drugs) designed to ignore the PTC and thereby produce a full-length PAX6 protein that does not degrade (8, 9). Likewise, the genetic background is a significant factor affecting the ocular phenotype. The Sey allele on the C57BL/6 genetic background results in significant variability, with moderate to severe microphthalmia and structural abnormalities; however, microphthalmia is seldom reported in aniridia. Moreover, mutations on C57BL/6 and BALB/c backgrounds result in an aggressive, advanced AAK phenotype apparent upon eye opening in the early postnatal period (6, 10, 11). This is a clear departure from the majority of human aniridia cases, where the cornea is transparent and presents with a very mild AAK that generally does not progress during the first decade of life (12). Consequently, a critical therapeutic window for aniridia that exists early in life in humans (1, 12) has been inaccessible for therapy development in mouse models.

The *Pax6*^+/–^ haploinsufficient Sey mouse model on the 129S1/SvImJ and hybrid F1 backgrounds was recently presented as an alternative aniridia model (11) that has a PTC mutation representing the most frequent human aniridia-causing mutation type. In particular, the mouse model on the 129S1/SvImJ background exhibited slower development of corneal neovascularization, but the full characteristics of the model on this background, including the ocular and AAK phenotype, are largely unknown. Here, we perform deep phenotype characterization of the dynamics of AAK development in this model, to evaluate its relationship to human disease. We provide a comprehensive analysis of the histologic, morphometric, and immunohistochemical features of the cornea in this mouse model with heterozygous *Pax6*^+/–^ genotype, relative to wild-type (WT; *Pax6^+/+^*) littermates, showing that the model mimics the human disease and provides a previously inaccessible potential therapeutic window for AAK, to facilitate detailed mechanistic and therapeutic investigations of relevance for human congenital aniridia.

## Results

### Pax6^+/–^ haploinsufficient 129S1/SvImJ heterozygous mice exhibit human-like, delayed-onset AAK phenotypes.

**Figure 1:**
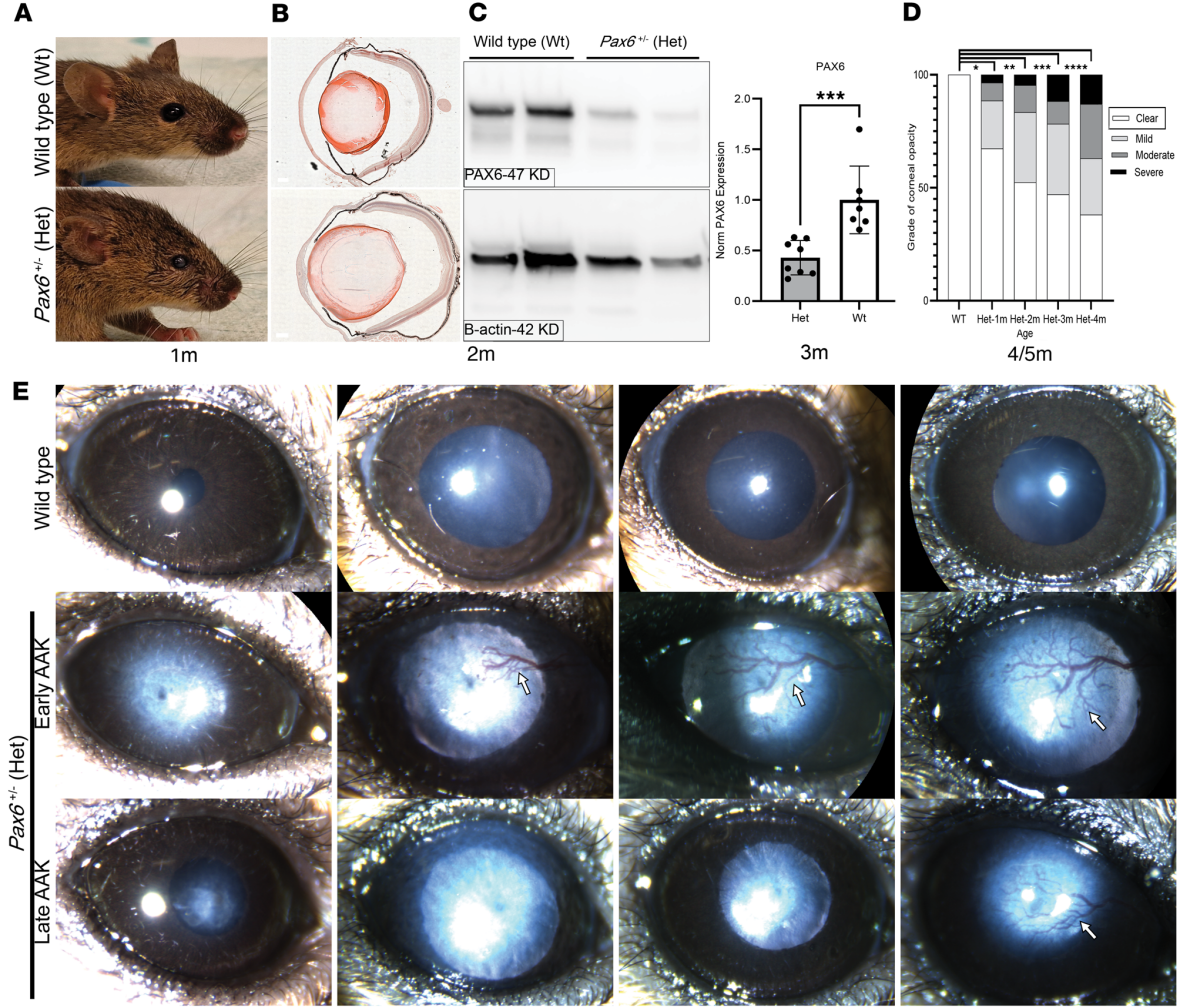
The *Pax6*^+/–^ 129S1/SvImJ mouse model phenotype mimics the variable onset of human AAK caused by PAX6 haploinsufficiency. (**A**) Representative images of a WT and Het mouse head showing only slightly smaller and less bulging eyes. (**B**) H&E staining reveals an underdeveloped anterior segment in Het mice. Scale bar = 200 μm. (**C**) Western blot analysis indicates a significant 58% decrease in PAX6 protein levels in corneas from Het mice compared with WT littermates, *n* > 7 (2-tailed *t* test ***P < 0.001). (**D**) Longitudinal characterization over a period of 5 months indicates a gradual and distributed pattern of loss of corneal transparency, *n* > 32 (Kruskal-Wallis test ****P < 0.0001, group comparisons * for *P* < 0.05, ** for *P* < 0.01, *** for *P* < 0.001, and ****P < 0.0001). (**E**) Slit lamp analysis revealed unchanged cornea status in WT mice and early and late AAK onset phenotypes in Het mice, both being progressive.

The heterozygous (Het) phenotype was visually discernible in adult mice upon observation, with smaller eye openings and more closed eyelids (Figure 1A). Relative to WT mice on the same background, hematoxylin and eosin (H&E) staining revealed defects in Het mice, such as keratolenticular adhesion, shallow anterior chamber, thickened cornea at the site of attachment, and iridocorneal angle abnormalities (Figure 1B). Examining the level of PAX6 protein, Western blot (Figure 1C) showed a PAX6 protein level of 42% in Het mice relative to WT (*P* < 0.001).

To assess AAK development, 26 Het mice and 24 WT mice were characterized longitudinally, with monthly examination up to 5 months using slit lamp biomicroscopy. In WT mice, a fully transparent cornea with full iris remained unchanged up to 5 months (Figure 1, D and E). In Het mice, the onset of AAK was variable and progressive, as assessed by the degree of central corneal opacity (loss of transparency). Central corneal opacity progressively increased with age in Het mice while WT corneas remained transparent (Figure 1D). In eyes exhibiting AAK (defined by the degree of blood vessel invasion into the cornea), 2 general phenotypes were apparent. In the early-AAK phenotype, invasion of blood vessels into the cornea occurred first at 1–2 months of age, gradually progressing from the periphery to the center of the cornea at 5 months of age (Figure 1E). In the second, late-AAK phenotype, vessel invasion was apparent first at 4–5 months of age (Figure 1E).

### Abnormalities in the cornea, lens, and iris in Het mice.

**Figure 2:**
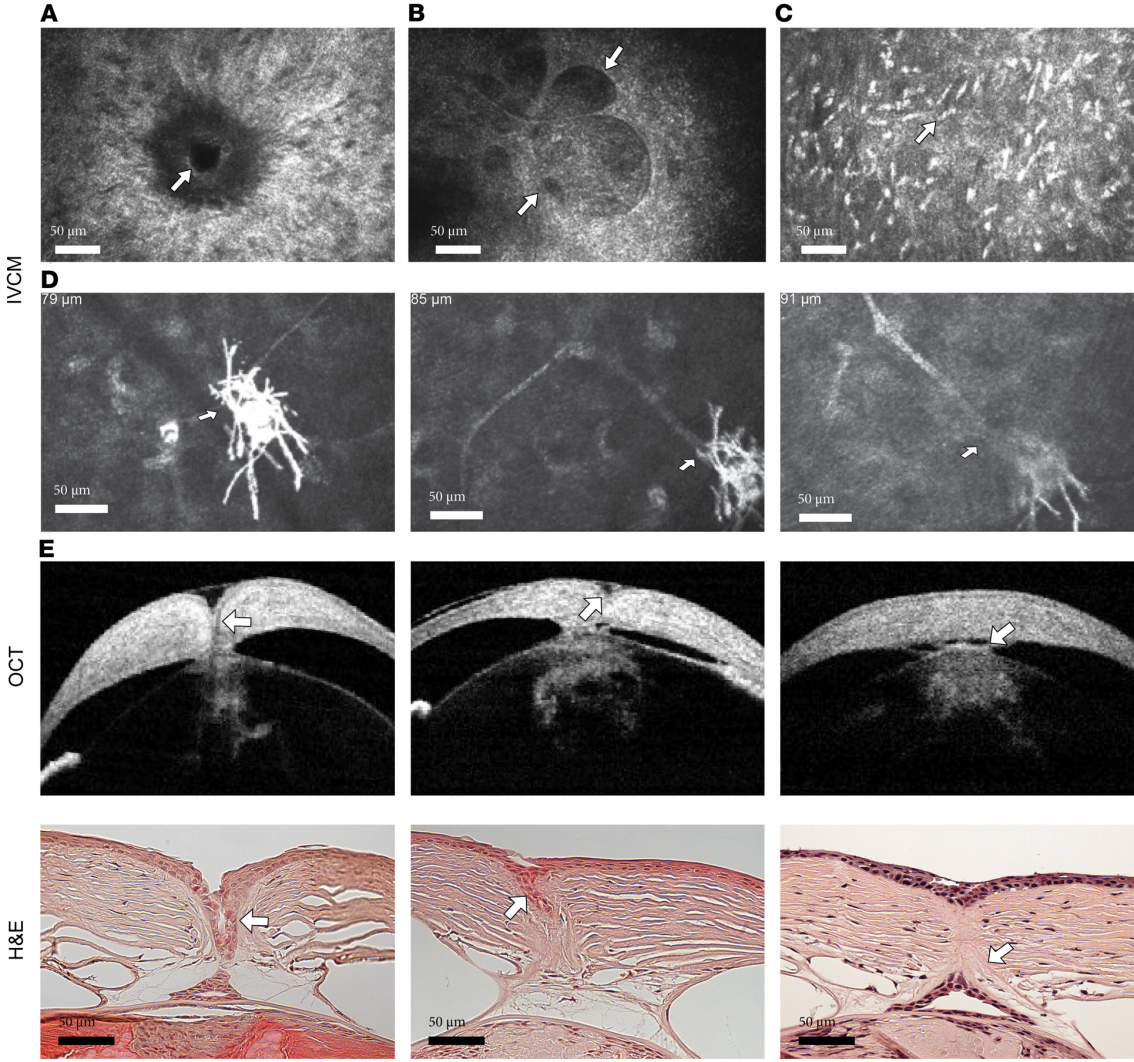
Corneal microstructural abnormalities in Het mice. (**A**) Point of keratolenticular adhesion in the posterior corneal stroma (arrow). (**B**) Dark vacuole structures (arrows) in the posterior corneal stroma. (**C**) Infiltration of inflammatory cells (arrow) in the corneal stroma. (**D**) Serial IVCM sections at different depths indicating the presence of a neuroma (arrows) and the attachment point to a stromal nerve trunk (arrows). (**E**) In vivo images of different degrees of keratolenticular adhesions by OCT (top row), with lens material indicated by arrows, and the corresponding H&E staining from the same eye, indicating the lens cells within the cornea (arrows, bottom row).

Table 1 provides a summary of the various abnormalities observed in the eyes of up to 60 Het mice by in vivo slit lamp and in vivo confocal microscopy (IVCM) examinations in mice of different ages. There was no significant sex difference noted in any of the parameters at the different ages (Supplemental Table 1; supplemental material available online with this article; https://doi.org/10.1172/jci.insight.183965DS1). The diverse frequencies of abnormal findings in Table 1 reflect the varied phenotypes induced by haploinsufficiency. However, keratolenticular (cornea-lens) adhesion was the most consistent feature observed in 100% of eyes in Het mice by IVCM examination, a feature observed even at a late prenatal stage (Supplemental Figure 4). The adhesion appeared in vivo as a dark ring structure in the posterior stroma (Figure 2A). In 15%–20% of eyes of Het mice, dark vacuole structures were visible in the posterior stroma near the keratolenticular adhesion (Figure 2B). Occasionally, this adhesion was minimal and challenging to detect with a slit lamp. However, further IVCM investigation verified the presence of adhesion (Supplemental Figure 1). Adhesions could also be confirmed by optical coherence tomography (OCT) examination, scanning the cornea in all directions. Infiltration of inflammatory cells into the corneal stroma was observed in up to 33% of eyes of Het mice at various ages (Figure 2C). The second most common feature observed in the cornea was neuromas, observed by IVCM imaging as a group of prominent nerve endings present at the termination point of a stromal nerve trunk (Figure 2D).

Iris coloboma was present in about half of corneas from Het mice at all ages, with variable degree and patterns of coloboma apparent (Supplemental Figure 2). The persistence of lens remnants within the cornea is depicted in histology and in vivo by OCT in the same eyes in Figure 2E. Separation of the lens vesicle from the surface ectoderm during fetal development was incomplete in Het mice, resulting in keratolenticular adhesions of various degrees.

### Het mice exhibit thinner corneas and a compromised epithelial barrier.

**Figure 3:**
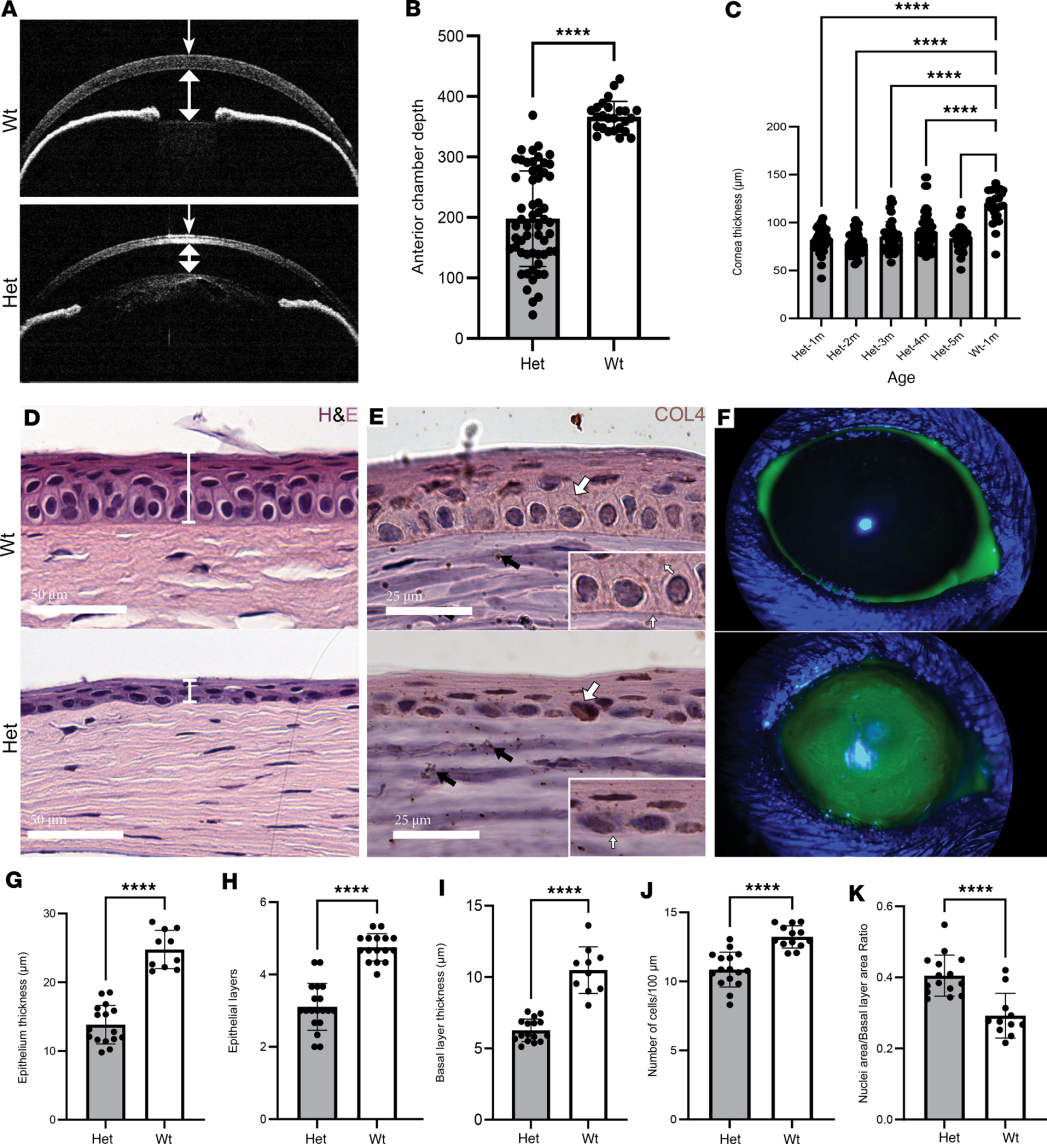
Corneal and epithelial structural abnormalities in Het mice. (**A**) OCT images of WT and Het mouse eyes indicating thinner corneas and shallower anterior chamber depth in Het mice. (**B**) Anterior chamber depth is reduced by half in Het mice; *n* > 25. (**C**) Central corneal thickness measured in vivo with OCT indicated significantly thinner corneas in Het mice not changing with age; *n* > 25. (**D**) Representative H&E images of corneas from Het and WT mice revealing thinner epithelium (marked) in Het mice. (**E**) COL4 DAB staining revealed absence of COL4 production in basal epithelial cells and absent epithelial basement membrane in Het mice (inset, white arrows), and dot-like expression of COL4 in the stroma representing basement membrane surrounding corneal nerves (black arrows) (original magnification, 20×; inset magnification, 28×). (**F**) Fluorescein staining indicated compromised epithelial barrier function with stromal uptake of the dye in vivo. (**G**–**K**) Comparative analysis from H&E images from Het (*n* = 16) and WT mice (*n* > 10). (**G**) Epithelial thickness, *n* > 10. (**H**) Number of epithelial layers, *n* > 17. (**I**) Basal layer thickness, *n* > 10. (**J**) Number of basal cells per 100 μm linear distance, *n* > 13. (**K**) Nuclear area/basal layer area ratio in basal cells, *n* > 11. In all panels **** indicates *P* < 0.0001 (2-tailed *t* test in 2-group panels or ANOVA multiple pairwise comparison).

OCT images revealed a fully developed anterior chamber with clear and consistent iridocorneal angle in WT mice, but markedly shallower anterior chamber depth in the eyes of Het mice, which was about half the depth of WT (*P* < 0.0001; Figure 3, A and B). Central corneal thickness was measured from OCT images of 235 eyes, with corneas of Het mice being 28.7% thinner than WT mice at 1 month of age (*P* < 0.0001), retaining this reduction up to 5 months (Figure 3C).

Histological investigation of the cornea revealed loss of columnar structure of basal epithelial cells, resulting in flattening of basal epithelial cells and loss of the distinctive contrast pattern of nuclear staining in corneas of Het mice relative to WT mice (Figure 3D). The corneal epithelium in Het mice also appeared markedly thinner than the WT corneal epithelium (Figure 3D). As basal cells build the epithelial basement membrane by producing and secreting components such as collagen IV (COL4), this was also examined. A distinct loss of collagen IV expression in the cornea of Het mice was noted, and an epithelial basement membrane was not discernible. Additionally, loss of collagen IV expression within the cytoplasm of basal epithelial cells was noted (Figure 3E). A dot-like expression pattern of collagen IV was apparent in the corneal stroma of WT mice, representing basement membrane surrounding corneal nerves (13), which was also visible in corneas of Het mice to some extent (Figure 3E). Staining of the live cornea with fluorescein dye and imaging under blue light confirmed impairment of the epithelial barrier function, with stromal uptake of the dye visible in corneas of Het mice (Figure 3F).

Quantitative analysis of H&E-stained sections verified that relative to the WT mice, the corneal epithelium in corneas of Het mice was half the thickness (*P* < 0.0001, Figure 3G). Additionally, the mean number of stratified cell layers in the epithelium was reduced from 5 in WT to 3 in Het mice (*P* < 0.0001, Figure 3H). Basal epithelial cell layer thickness was likewise reduced in Het mice (*P* < 0.0001, Figure 3I). Basal epithelial cell density in Het mice was also reduced, with a significant reduction in the number of basal cells per 100 μm of linear distance (*P* < 0.0001, Figure 3J). The nuclear area/basal layer area ratio was significantly increased in Het mice, with nuclei comprising most of the cellular volume (Figure 3K).

### Het mice exhibit delayed onset and variable progression of AAK.

**Figure 4:**
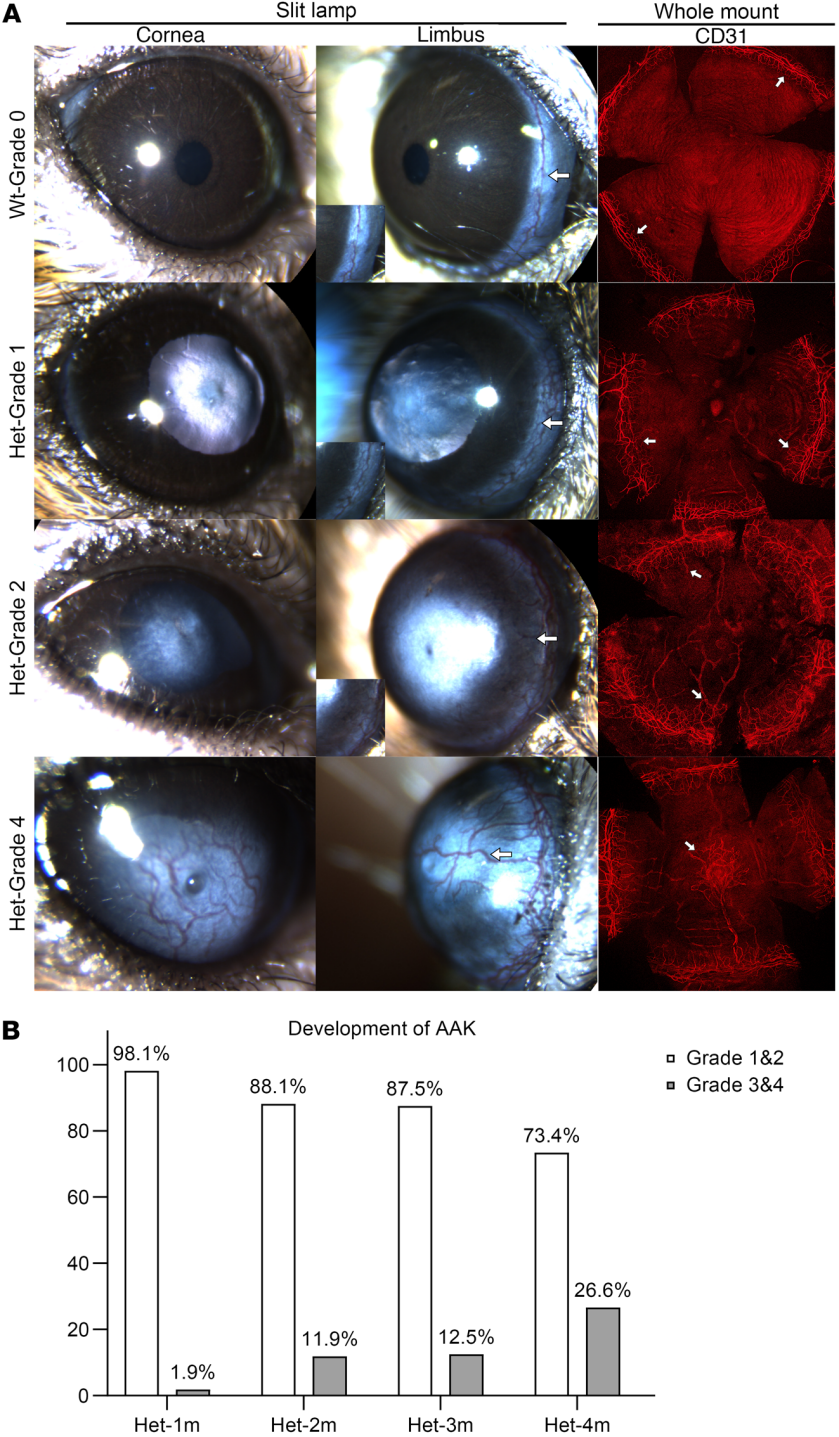
Grade and progression of AAK in Het mice. (**A**) Representative slit lamp images from 1 or 2 mice of each grade and whole-mount staining with CD31 for each grade, indicating different grades of AAK. In WT mice, arrows indicate the intact limbal border. In Het mice, arrows indicate blood vessels that have just breached the limbal border and entered the cornea. (**B**) Distribution of AAK grade among Het mice of different ages, *n* > 32.

Progression and severity of AAK were assessed by slit lamp biomicroscopy to visualize the limbal region in all mice, applying the clinical grading scale for AAK (Figure 4A). In the eyes of WT mice, a transparent cornea with intact limbal border was present, and whole-mount staining with CD31 verified vessels were confined to the limbal border, representing grade 0 AAK. In Het mice at 1 month of age, the majority (98%) had grade 1 or 2 AAK with a transparent central cornea but with vessels just breaching the limbal border and entering the peripheral cornea. As Het mice aged, AAK progressed with vessels entering the central cornea (grade 3) and eventually resulting in a thick, white vascularized tissue covering the entire cornea (grade 4). Relative proportions of Het mice with different AAK grades at different ages are given in Figure 4B. Notably, a large proportion of Het mice remained in early-stage grade 1–2 AAK as they aged.

### Progressive breakdown of the corneal cellular microenvironment in AAK.

**Figure 5:**
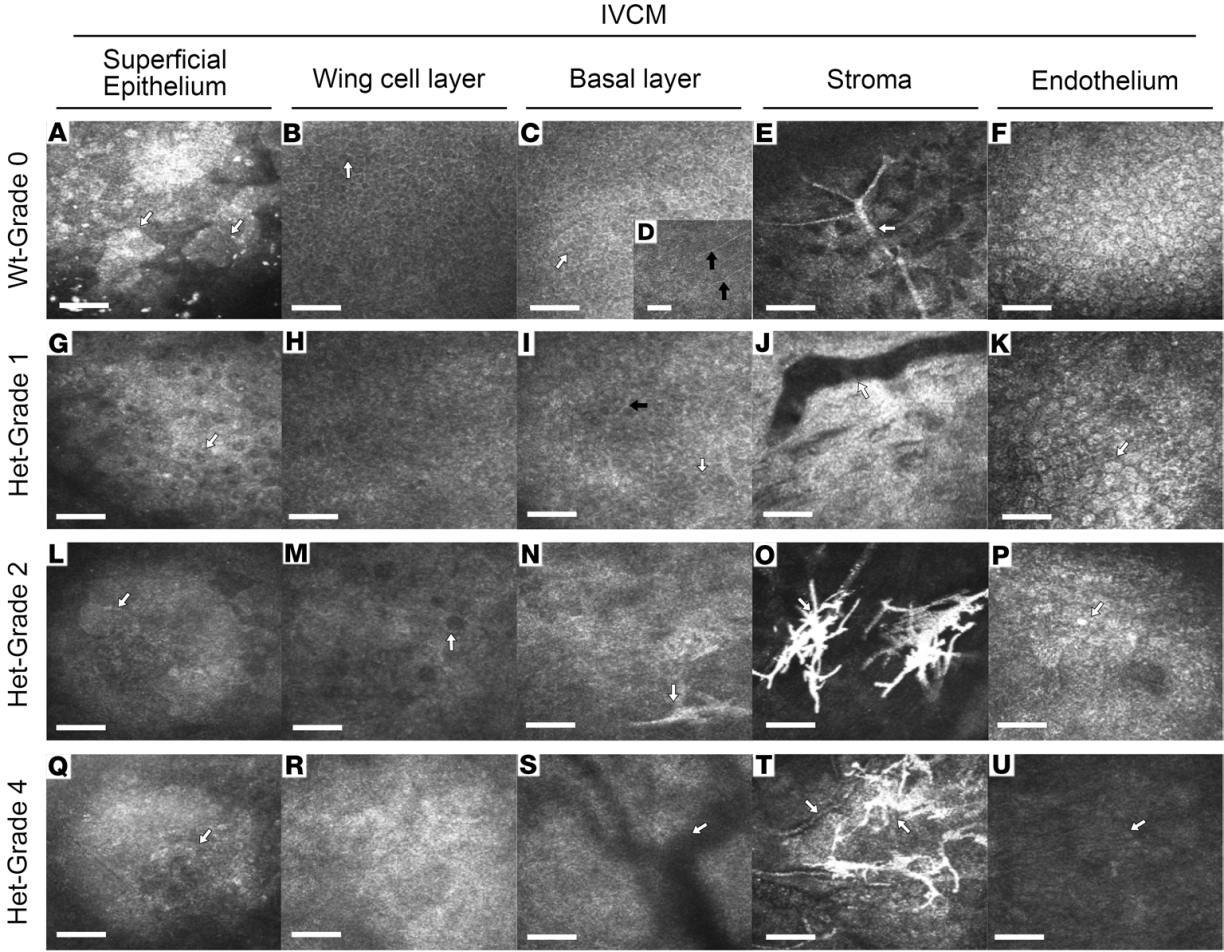
Longitudinal in vivo examination of corneal microstructure with IVCM. (First row) Normal corneal cellular layers in WT mice. (**A**) Polygonal flat (arrow) superficial epithelial cells. (**B**) Wing cell layer with bright borders and dark cytoplasm (arrow). (**C**) Basal cell layer with bright borders and densely packed cells (arrow). (**D**) Network of subbasal nerves within a dense nerve plexus (black arrows). (**E**) Branching stromal nerve fiber trunk (arrow). (**F**) Hexagonal monolayer of endothelial cells. (Second row) Cornea layers in grade 1 AAK. (**G**) Superficial cells are small, nonpolygonal, and with dark nuclei (arrow). (**H**) Loss of distinct mosaic pattern and loss of dark cytoplasm of wing cells. (**I**) The parallel and regular pattern of subbasal nerves is disrupted (black arrow). (**J**) Vascular structure in the stroma. (**K**) Visible endothelial cells (arrow). (Third row) Cornea layers in grade 2 AAK. (**L**) Indistinct superficial epithelial cells (arrow). (**M**) Dark vacuole-like structure (arrow) and loss of cellular mosaic. (**N**) Loss of distinct cellular structure, with neuroma visible that approaches the basal layer (arrow). (**O**) Large hyperreflective neuromas (arrow) in stroma. (**P**) Indistinct hexagonal endothelial cell (arrow). (Fourth row) Cornea layers in grade 4 AAK. (**Q**) Very small superficial cells (arrow). (**R**) Fibrous tissue replacing the wing cell layer. (**S**) Shadows of stromal vessels (arrow) at the level of the basal layer. (**T**) Coappearance of vessels and neuromas in stroma (arrows). (**U**) Faintly detectable nuclei (arrow) of endothelial cells. Scale bars = 50 μm.

To investigate the impact of AAK development on the cornea at the microscopic level, longitudinal in vivo imaging of mouse corneas was performed using IVCM. Epithelium (superficial, wing, and basal layers), stroma, and endothelium were imaged (Figure 5, A–F). The large-area flat, polygonal, superficial epithelial cells with bright nuclei in WT mice (Figure 5A) were replaced by smaller, oval-shaped cells with dark nuclei in Het mice and became progressively smaller and less distinct as AAK progressed (Figure 5, G, L, and Q). Wing cells that normally have a distinct mosaic pattern with clear cell borders and dark cytoplasm and no visible nuclei (Figure 5B) were transformed in grade 1 AAK to cells with bright nuclei, having lost the mosaic pattern and distinct cell borders (Figure 5H), which in more advanced AAK grades completely lost all cellular structure and exhibited vacuoles (Figure 5M) and fibrous material (Figure 5R). The basal cell layer of WT mice with densely packed cells with bright cell borders and dark cytoplasm (Figure 5C) with visible nerves of the subbasal epithelial nerve plexus (Figure 5D) was transformed in grade 1 AAK of Het mice to a cell layer with bright nuclei and indistinct cell borders, with disrupted subbasal nerve fiber orientation (Figure 5I) that in later stages lost all cellular structure (Figure 5, N and S). Bright (hyperreflective) nuclei of stromal keratocytes and thick stromal nerve fiber trunks (Figure 5E) in WT mice were no longer visible in corneas of Het mice, instead being replaced by fibrovascular tissue with abnormal microneuromas (Figure 5, J, O, and T). A consistent monolayer of endothelial cells was visible in the normal corneal endothelium (Figure 5F) and in the endothelium of Het mice, but this posterior layer became more difficult to discern with increasing AAK grade (Figure 5, K, P, and U). Taken together, the results indicate a progressive breakdown of the cellular and neural structure of the corneal epithelium and stroma in Het mice that is discernible at the earliest stage in grade 1 AAK when the central cornea is still transparent.

### Het mice exhibit selective and persistent postnatal corneal lymphangiogenesis.

**Figure 6:**
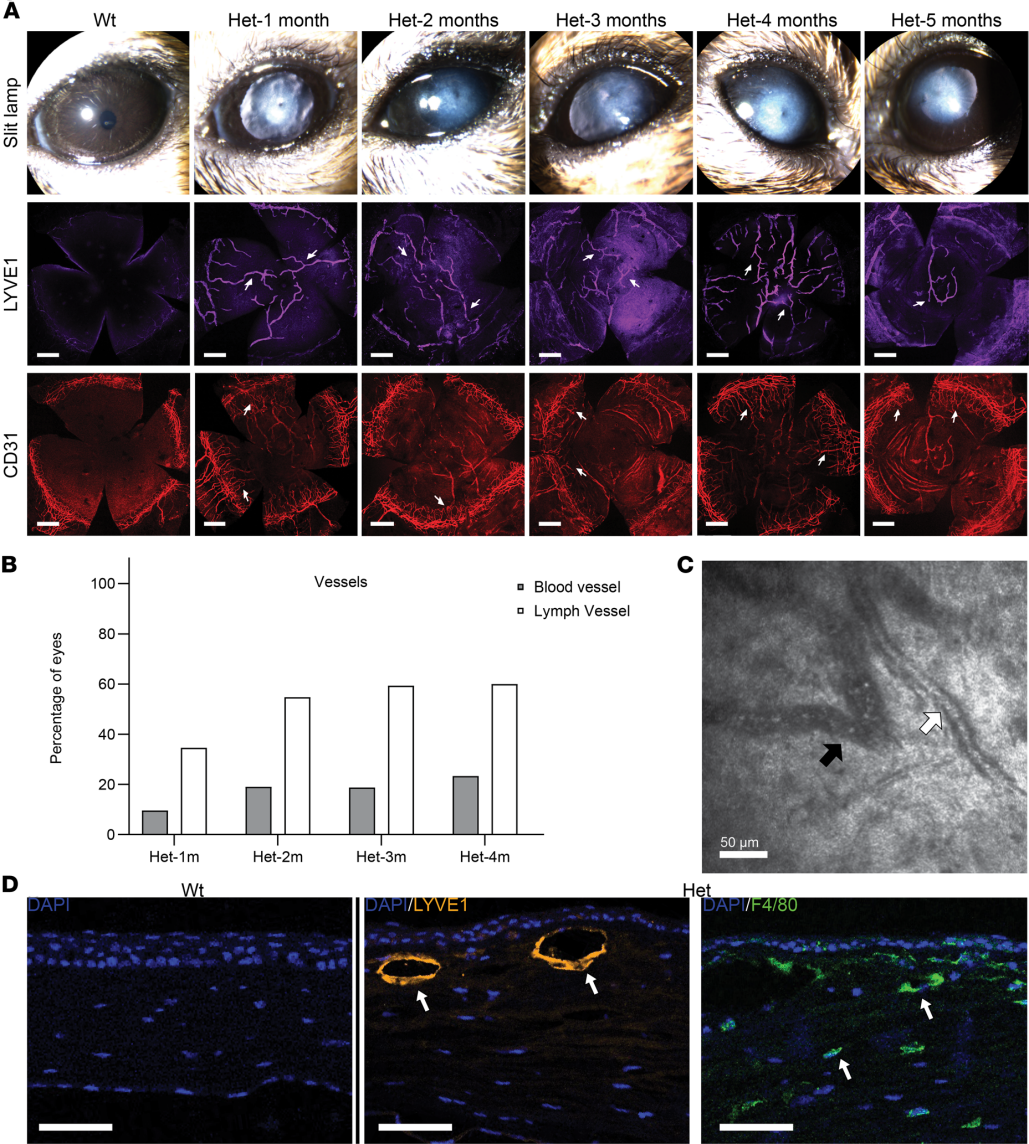
Time course and characteristics of corneal neovascularization. (**A**) Slit lamp images (top row) confirming grade 1 and 2 AAK in Het mice up to the age of 5 months. Corresponding whole-mount corneas immunostained for lymph vessels (LYVE1, middle row) and blood vessels (CD31, bottom row). White arrows in both rows highlight lymph and blood vessels, respectively. Scale bar = 500 μm. (**B**) Prevalence of corneal lymph vessels was 3–4 times higher than that of blood vessels at all ages, *n* > 32. (**C**) IVCM image indicating the presence of lymph vessel (black arrow) and blood vessel (white arrow) at the same stromal depth. (**D**) Immunostaining of cornea sections revealed LYVE1-positive vessels (orange) and F4/80^+^ (green) cells throughout the stroma, verifying the coincidence of macrophage infiltration and lymphangiogenesis in Het but not in WT mouse corneas. Scale bars = 50 μm.

To characterize corneal neovascularization with AAK development as an indicator of progressive LSC deficiency, whole-mount corneal tissues were immunostained for blood (CD31) and lymphatic (LYVE1) vessels. Surprisingly in Het mice, an early and selective ingrowth of corneal lymph vessels was observed at 1 month of age, extending into the central cornea even without the presence of blood vessels (Figure 6A). These lymphatics did not regress but persisted in the cornea to at least 5 months of age. CD31 staining revealed that corneal hemangiogenesis was less prominent and delayed relative to corneal lymphangiogenesis. In WT mice, however, the cornea maintained corneal angiogenic privilege, devoid of both blood and lymphatic vessels (Figure 6A).

Blood and lymph vessel frequency was tracked over time in mice (Figure 6B), revealing that lymph vessels were highly prevalent in the corneas of Het mice regardless of age, and appeared to be independent of blood vessel presence, indicating that lymph vessels are more dominant than blood vessels in Het mouse corneas and persist over time.

In the IVCM image depicted in Figure 6C, lymphatic vessels were observed coexisting with blood vessels at the same corneal depth. These lymphatic vessels appear morphologically distinct from adjacent blood vessels and are characterized by a larger and irregular diameter compared with blood vessels. In addition, lymph vessels have no discernible vessel walls, a dark lumen that is consistent with the transparency of the lymph fluid, and a few visible reflective cells (presumed leukocytes) compared with blood vessels that exhibit a smaller vessel diameter, thicker linear vessel walls, and a high number of small reflecting cells (erythrocytes) (14).

Histological sections from the neovascularized area of a 2-month-old Het mouse revealed LYVE1-positive vessels in the anterior corneal stroma, with additional F4/80 staining of macrophages throughout the stroma. By contrast, WT mouse corneas were devoid of both LYVE1- and F4/80-positive cells (Figure 6D).

### Disrupted corneal nerve organization in Het mice.

**Figure 7:**
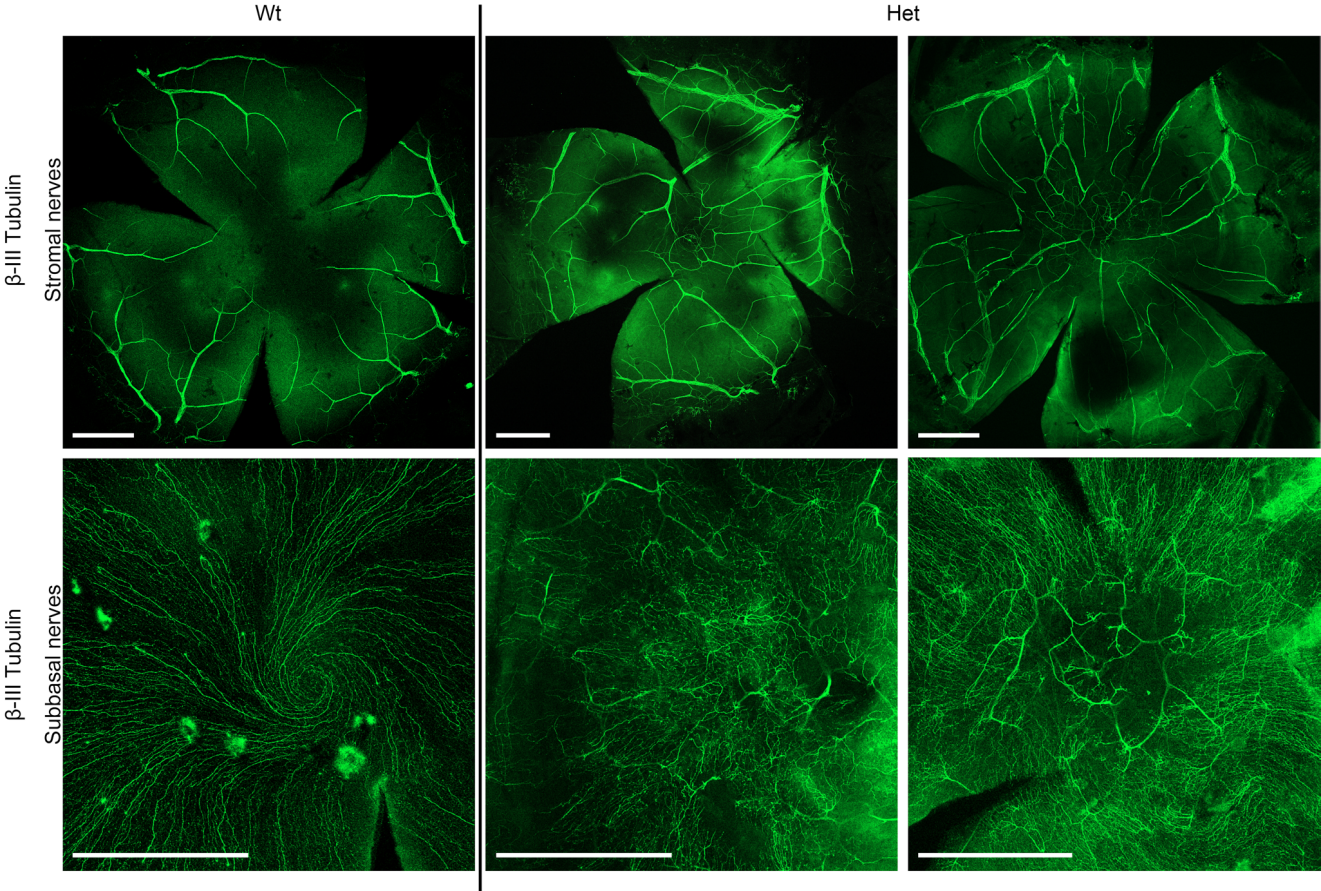
β-III tubulin immunostaining of corneal nerves in whole-mount corneas. (Top row) Stromal nerve network, with central stromal nerves being scarce in WT mice and highly prevalent in Het mice. (Bottom row) Vortex pattern of subbasal nerves in WT mice and disrupted pattern of subbasal nerves in Het mice with anterior stromal nerves invading the subbasal layer. Scale bars = 500 μm.

β-III tubulin whole-mount immunostaining of corneal nerves (Figure 7) revealed that in WT mice, stromal nerves were mainly confined to the peripheral cornea with very few stromal nerves in the central cornea. In contrast, in corneas of Het mice, stromal nerves extended into the central cornea (Figure 7). Epithelial nerve bundles derived from the peripheral stromal nerve branches form a dense nerve layer called the corneal subbasal nerve plexus (SBNP). In corneas of WT mice, nerve fibers in the SBNP were densely distributed and organized centripetally, converging to form a vortex at the corneal apex. In Het mice, this architecture was disrupted, with an uneven and sporadic distribution of nerves in the SBNP that did not exhibit any clear spatial pattern. Moreover, in the central cornea, anterior stromal nerve trunks invaded the SBNP layer in the corneas of Het mice, indicating loss of stromal-epithelial compartmentalization of nerves (Figure 7).

### LSC markers persist in the basal epithelium, and remnants of stem-like lens epithelium persist in the stroma of Het mice.

**Figure 8:**
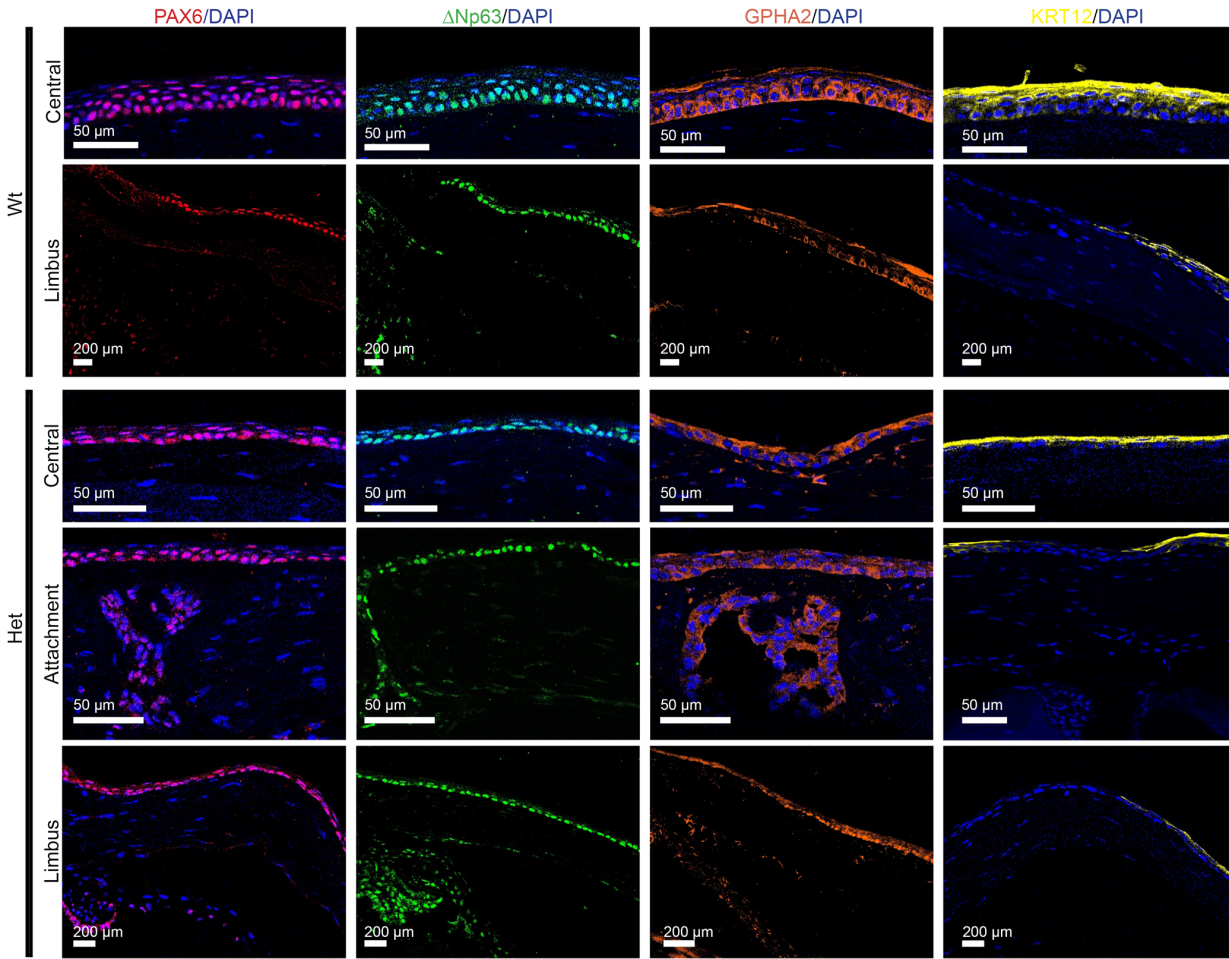
Immunolocalization of PAX6, ΔNp63, GPHA2, and KRT12 in the center, limbus, and central attachment zone in cross sections of corneas from WT and Het mice with grade 1 and 2 AAK. (First column) PAX6. (Second column) ΔNp63. (Third column) GPHA2. (Fourth column) KRT12. Nuclei are counterstained with DAPI (blue) in most sections.

As the emergence of AAK coincides with a progressive limbal stem cell deficiency (LSCD) in human aniridia (15), the location and distribution of LSCs and differentiated epithelial cells were investigated along with PAX6 expression in Het corneas in grade 1 and 2 AAK (Figure 8). In WT mice, PAX6 was strongly expressed in all epithelial layers while in Het mice, PAX6 expression was confined mainly within the flattened basal layer of the thin epithelium. PAX6 expression continued to the limbal basal epithelium, where it was expressed equally and uniformly in the basal cells of the limbus in WT and Het mice. The basal epithelium strongly expressed LSC marker ΔNp63 and the putative LSC marker GPHA2 in the central and limbal cornea of both WT and Het mice. The superficial epithelial layer of the central cornea strongly expressed the KRT12 differentiation marker in WT and Het mice (and notably did not strongly express PAX6 or stem cell markers). Superficial epithelium, however, lacked KRT12 expression in the area of keratolenticular attachment in Het mice and in the limbus of both WT and Het mice. The expression of PAX6, ΔNp63, and KRT12 showed a similar pattern in grade 3 and 4 AAK; however, GPHA2 in these more advanced AAK stages shifted to the superficial epithelium (Supplemental Figure 3). The proportion of Ki-67–positive cells was elevated in the basal layer of corneal epithelia in Het mice compared with normal WT tissue and became more prominent as AAK developed to grades 3 and 4. In addition, many MUC5AC-positive cells were detected in the epithelia of Het mice with later stage AAK, exhibiting a uniform MUC5AC expression throughout the epithelium, whereas MUC5AC expression was absent in the epithelia of WT mice (Supplemental Figure 5).

Interestingly, within the central cornea of Het mice where the keratolenticular attachment is present, clusters of cells were embedded within the corneal stroma anterior to the lens capsule. These clusters exhibited a morphology distinct from the typical flattened nuclei of corneal keratocytes. The positivity for stem cell markers (ΔNp63 and GPHA2) and the absence of KRT12 expression within these stromal clusters imply the presence of a stem-like cell population originating from the keratolenticular adhesion.

## Discussion

AAK has been identified in almost 80% of patients with congenital aniridia (2), while the prevalence of a minimal keratopathy at the microscopic level has been reported to be 100% in aniridia (7). AAK in human aniridia leads to substantial visual morbidity, and importantly, there are no approved pharmacological treatments targeting its pathogenesis. An animal model that closely mimics the qualities of human AAK would facilitate deeper investigations of pathophysiological mechanisms and enable evaluation of potential therapies, which have been previously tested in mouse models exhibiting an advanced AAK (with malformed eyes) at birth (6, 10, 11), which makes results difficult to translate to the human disease. Here, advanced in vivo imaging modalities (IVCM and OCT) alongside histopathological examination were used to extensively document the dynamic structural alterations in the cornea at up to 5 time intervals in the *Pax6*^+/–^ Sey mouse model on the 129S1/SvImJ background, a model that uniquely mimics the slow onset and progression of AAK that is not seen in other Sey mouse models.

In humans the natural course of AAK usually begins after the first decade of life and progresses with age (7, 12, 15, 16), a timeline similar to Het mice in the present study, which importantly provides a window of up to several months in mice where the central cornea remains transparent prior to AAK progression. Other advantages of the present model are the time of AAK emergence and rate of AAK progression. These, along with the bilateral asymmetry of AAK and even the variation in phenotype across individuals, all align with various clinical observations of AAK in humans (7, 16). An association of AAK severity with age was found in the characterized Het mice, notably with no eye having grade 0 upon careful slit lamp examination. By 1 month of age, blood vessels were detected centrally or paracentrally in only 1.9% of eyes of Het mice, increasing to 26.6% of eyes by the age of 4 months. Grade 1 and 2 AAK were more prominent in younger mice, while older mice mainly exhibited grade 3 and 4 (Figure 4). This corresponds to the human AAK development, where patients under 20 years of age are mostly reported with grade 1 AAK while those older than 20 years usually progress to grades 2–4 (16). Our findings also demonstrated a direct correlation between the extent of corneal opacification and the level of peripheral vascularization as the Het mice age, consistent with observations made in humans.

Although the abnormality of the epithelium in aniridia has previously been documented, here we quantify several aspects of the compromised epithelium in the present mouse model, that are further discussed below. At the histological level, depletion of collagen IV, a prominent constituent of basement membranes, was notable in Het mice. A similar reduction in collagen IV has been previously reported in a patient with aniridia (17), and loss of basement membrane has been noted histopathologically in humans (18). The deficiency in collagen IV is expected to lead to absent or aberrant basement membrane structure, disorganization of the basal layer, and a diminishment of cellular demarcations, leading to fragility of the epithelium. This finding was corroborated by the present in vivo findings using IVCM, where the cell borders and cellular mosaic structure diminished in conjunction with advancing AAK grades. As the basement membrane provides mechanical support, divides tissue into different compartments, and influences cell proliferation, differentiation, and migration, the compromised basal epithelium and basement membrane in Het mice, together with deficiency in the underlying collagen IV matrix structure, may result in a decline in epithelial-stromal barrier function. This observation was verified by the infiltration of fluorescein dye, consistent with findings from Ou et al.’s investigation on a *Pax6*^+/–^ mouse model (19). We note here also that this function may be closely tied to the sparse, degenerate phenotype of the basal epithelial cells that no longer appear to produce collagen IV. Moreover, presumably due to this loss of epithelial-stromal barrier, stromal nerves migrated beyond the stromal compartment to invade the central subepithelial space, replacing the normal dense spiraling network of subbasal nerves in the central cornea with less dense and disorganized stromal nerve trunks. Moreover, abnormal hyperproliferative stromal nerve endings called microneuromas, not detected in the corneas of WT mice, were observed in close proximity to the epithelium in Het mice, further suggesting a disruption of the nerve-epithelium homeostasis. Loss of the subbasal nerve spiral pattern, projection of stromal nerves into the epithelium, and presence of dense “knotting” of nerves (microneuromas) were all noted previously by Leiper et al. in a Sey-Neu aniridia mouse model (20), suggesting a compromised neurotrophic status as also observed in the present model. This closely resembles the declining subbasal nerve density with age reported in human studies (15, 21). We note here that the neurotrophic deficit is already present in grade 1 AAK, comparable to the early stages of AAK in patients (22). Moreover, the related early and progressive changes in the epithelial wing and basal cell layers noted here by IVCM with increasing AAK severity have also been reported in patients with aniridia (17).

Taken together, the compromised epithelial function and structure, along with disturbed nerve organization and infiltration of inflammatory cells observed in this model, are aligned with the hypothesis that perturbations in ocular surface homeostasis disrupt the vital interaction between nerves and epithelial cells. This results in disruption of the normal corneal renewal process, leading to an inflammatory cascade and compromised wound healing, tipping the balance toward neovascularization by shifting expression of antiangiogenic factors to proinflammatory and regenerative factors (1), also seen in human cases of aniridia (4, 23).

Among inflammatory cells infiltrating the cornea, macrophages are potent triggers of lymphangiogenesis because of the secretion of vascular endothelial growth factors C and D and their physical incorporation within forming lymphatics (24). In the present model, immunostaining revealed corneal lymph vessels accompanied by stromal infiltration of F4/80^+^ macrophages. Intriguingly, we also report the currently undocumented, selective, and early invasion of LYVE1^+^ lymphatics into the central cornea without the presence of CD31^+^ blood vessels, which persisted over time in Het mice, without natural regression of these vessels. This finding is notable given that the normal mouse cornea is endowed with a significant number of lymphatic vessels during development, which subsequently undergo spontaneous regression to the limbal area after eye opening (25). The persistence of these vessels in Het mice provides further evidence for the hypothesis of a frozen ocular developmental state in aniridia, which also encompasses incomplete normalization of corneal thickness, dendritic cell density, endothelial cell density, and corneal innervation (21). This suggests that inadequate levels of PAX6 could lead to the failure of spontaneous lymphatic regression and thus failure of the associated postnatal corneal immune privilege. Interestingly, this failure of lymphatic regression may be related to the persistence of proangiogenic macrophages (24) that were observed here within the stroma of Het mice. These macrophages, chronically present, may also over time contribute to blood vessel invasion of the cornea and thus progression of AAK; however, given the persistent inflammation, neurotrophic deficit, and compromised epithelium, multiple factors likely contribute to the progression of AAK.

AAK is often considered a progressive form of LSCD. Immunostaining in the present model showed qualitatively that fewer epithelial cells expressed LSC markers in Het mice than in WT. Thus, it is plausible that a reduced number of LSCs are developmentally determined in Het mice; yet, interestingly, the results also show that these cells have the ability to survive for extended periods and continue to express stem cell markers. However, these putative LSCs might be less efficient than WT LSCs in terms of proliferation and producing active, migrating progeny. In vivo studies have also provided evidence that limbal epithelial stem cells are likely preserved in human AAK (18). Moreover, visible palisades of Vogt structures in the early stages of AAK have been reported, which, along with preserved corneal epithelial phenotype in early stages, suggest a degree of preserved LSC niche function (15, 16, 22).

Surprisingly, immunostaining results from Het mice with late-stage grade 4 AAK also revealed the persistence of LSC markers in the epithelium. ΔNp63, a well-known marker for LSCs, was detected in the basal layer and suprabasal epithelial layer in WT mice, while in Het mice, ΔNp63 expression was confined to a single layer of flattened basal cells. In grade 4 AAK, further loss of ΔNp63 expression was apparent. Schlötzer-Schrehardt et al. proposed that these cells may represent hyperproliferative transient-amplifying progenitors, which ensure a continuous provision and swift turnover of epithelial cells that in aniridia are inadequately and aberrantly differentiated (18).

GPHA2 has been introduced as a new marker for quiescent stem cells, which are progenitor and slow-cycling cells surrounded by their progeny of abundant and fast-cycling cells (26, 27). It has also been shown that GPHA2 expression depends on niche-specific signals whose function appears to be essential for LSC self-renewal and differentiation (28). In WT mice, GPHA2 was expressed in the basal epithelial layer, while in Het mice, expression shifted toward superficial layers of the epithelium, which was even more prominent in grade 4 AAK, where expression in the basal epithelial layer was notably absent. Thus, GPHA2 expression in the basal epithelium appears to mirror the progressive limbal insufficiency in AAK and could be an important marker for further studies.

The increased expression of Ki-67 in the corneal epithelium in Het mice suggests heightened proliferation of progenitor cells within the basal layer, contributing to an accelerated turnover of epithelial cells that are poorly differentiated, as noted above from the pattern of ΔNp63 expression. This leads to the development of conjunctival and epidermal phenotypes evidenced by MUC5AC staining of epithelial cells found in Het mice that increased as AAK progressed. Similar MUC5AC expression was found in the context of human AAK (18). Previous human studies of AAK have also demonstrated a clear transformation of corneal to conjunctival epithelium along with goblet cell invasion into the cornea (17, 29). This suggests that the epithelium in the Het mice is abnormally proliferative and transforms into conjunctival epithelium that recapitulates the phenotype and progression of human AAK.

Of additional note, the pattern of LSC marker expression in Het mice corresponded with our in vivo findings of progressive corneal opacification and vascularization associated with LSCD. As observed in slit lamp images in younger mice with grades 1–2 AAK, corneal transparency is maintained, implying at least partial function of the limbal niche and LSCs. As AAK progressed, LSC function was compromised, resulting in limbal barrier breakdown and invasion of blood vessels into the central cornea. Additionally, our IVCM results showed the increasing severity of LSCD correlated with a loss of normal corneal epithelial cell morphology and a decline in central subbasal nerves, in line with human studies (15, 30).

Whether the LSC function is compromised by the presence of other pathology, such as inflammation, neurotrophic deficit, and the presence of pro-angiogenic factors is unknown; however, marker expression in Het mice suggests a possibility for LSC survival, potentially even in late-stage AAK. The potential for maintaining or possibly even reversing the stem cell–related changes in AAK requires further exploration, and the Het mice characterized here provide an excellent model for future investigations of LSC biology in the context of *PAX6* transcriptional regulation. Importantly, current therapeutic interventions for aniridia aimed at restoring the niche environment to mitigate the deterioration of limbal epithelial stem cell function are solely surgical, and these have proven largely unsuccessful (3, 4).

Interestingly, in the region of corneas in Het mice in apposition with the keratolenticular attachment, clusters of cells were observed embedded within the corneal stroma. These cells exhibited morphological features distinct from corneal keratocytes and expressed PAX6, ΔNp63, and GPHA2, but not KRT12, and are presumed lens epithelial stem-like cells, which may originate from the developing prenatal lens.

In summary, the present Het mouse model is highly analogous to human aniridia and is the only model presented to date with slow-onset AAK as observed in humans, with the cornea remaining transparent for periods of up to several months. This mirrors the human AAK time frame of progression according to the corresponding life cycle of mice. Phenotypic and histological analyses verified that the complications observed in human AAK, such as a shallow anterior chamber, epithelial fragility, absence of collagen IV in the epithelial basement membrane, and retained hyperproliferative progenitor cells with subsequent maldifferentiation of epithelial cells, are accurately recapitulated in this mouse model. In addition, we note several findings in this model suitable for deeper investigation, such as altered basal epithelial structure and function, persistent and selective lymphangiogenesis, disrupted corneal nerve organization with microneuromas, and epithelial cells with partially retained stem cell markers even in late-stage AAK.

There remain, however, important discrepancies between our model and human AAK, namely thinner corneas in mice and consistent presence of a keratolenticular adhesion. The thin cornea is common across all mouse models of PAX6 deficiency (6, 10, 11, 31). This differs notably from the thicker corneal stroma in human AAK, which has been postulated to be a consequence of incomplete prenatal stromal development (21). The reason for this discrepancy between mice and humans is unclear and requires further investigation. The keratolenticular adhesion, present in all Het mice, results from incomplete separation of the cornea and the lens during prenatal development and has been observed in other small-eye mouse models (9, 10, 31, 32). The adhesion induces a localized loss of stromal transparency at the place of attachment evident even in young mice where AAK is not yet apparent and where the rest of the cornea is transparent. The adhesion did not appear to affect the timing or progression of vascular pannus ingrowth into the cornea or the grade of keratopathy, which was notably milder than in other small-eye mouse models. The small size of the adhesion, however, permitted examination of all aspects of the cornea and did not inhibit its characterization.

In summary, the present mouse model provides the potential to elucidate diverse pathophysiologic mechanisms in the most prevalent PAX6 mutation type observed in congenital human aniridia. Phenotypic features in the Het mouse cornea can provide objective endpoints for future translational studies, while importantly, the observed slow onset of AAK provides a relevant therapeutic window for future evaluation of targeted interventions.

## Methods

### Sex as a biological variable.

Both male and female mice were examined, and phenotypic findings are reported for both sexes combined and separately (Table 1 and Supplemental Table 1).

### Animal models.

In this study we characterized mice of the 129S1/SvImJ background. Specifically, the heterozygous 129S1.Cg-Pax6Sey/Mmmh strain (Mutant Mouse Resource and Research Center stock number 050624-MU) with a heterozygous *Pax6* mutation (11) was bred and genotyped at the animal facility of Linköping University to obtain homozygous nonmutant (*Pax6*^+/+^, WT) or heterozygous *Pax6* Sey mutant (*Pax6*^+/–^, Het) mice. Specifically, this mouse strain bears a spontaneous point mutation in the *PAX6* gene at exon 8 (nucleotide 27,726 on ENSMUSG00000027168, PAX6 mRNA nucleotide 903: G to T substitution) generating a premature stop codon. For colony-breeding purposes, *Pax6*^+/+^ females were bred with *Pax6*^+/–^ males. Mice were housed at 23°C and 40%–60% humidity on a 12-hour light/12-hour dark cycle. In total, 88 mice were characterized at various age stages, comprising 26 Het females, 16 WT females, 24 Het males, and 22 WT males. Experiments were in accordance with guidelines of the regional ethics committee for animal experiments at Linköping University, Sweden, and in line with the Association for Research in Vision and Ophthalmology guidelines for the use of animals in ophthalmic and vision research.

### Mouse genotyping and primer design.

To identify the mutational status of the mouse colony offspring and assign them to study groups, PCR was performed on genomic DNA from digested ear clip tissue taken at weaning. To identify the single point mutation, 2 pairs of primers were designed and used per sample: They included a common forward (FW) primer for both pairs upstream of the mutation site and 2 reverse (RV) primers with an intentional penultimate base mismatch (same in both RV primers, T instead of C) and an ultimate base that matched either the WT or the Sey allele (T or A, respectively). This, in the case of WT mice, resulted in a functional WT RV primer with 1 mismatch, and a nonfunctional Sey RV primer with a 2-base mismatch at the 3′ end, giving a product only in the FW/WT Sey primer pair. Conversely, in the case of Het Sey mice, the FW/WT RV pair gave a product from the normal allele, and the FW/Sey RV pair also gave a product from the mutated allele. Specifically, the primers used were common FW: CTGTGCCGAGTCCCATTAGG, WT RV: CTGAGCTTCATCCGAGTCTTCTTC, and Sey RV: CTGAGCTTCATCCGAGTCTTCTTA. DNA was extracted with 200 μL 50 mM NaOH digestion at 90°C followed by pH neutralization by 35 μL Tris-HCl (pH 8). DNA was measured and 150 ng of total DNA was loaded in each reaction, along with a final C of 0.5 μM of each primer in a reaction volume of 25 μL. We used 12.5 μL of 2× DreamTaq PCR Master Mix (Life Technologies, Thermo Fisher Scientific, K1072) in each reaction that ran for 35 cycles of 95°C, 60°C, and 72°C.

### Animal anesthesia and recovery.

During the characterization experiments, animals were anesthetized with intraperitoneal ketamine-xylazine injection (65 mg/kg ketamine, 10 mg/kg xylazine in a PBS dilution for 10 μL/g of weight injection), and topical anesthesia of tetracaine-HCl 0.5% drops was also applied. At the end of the characterization, if the time point was not terminal, mice were administered 1.8 mg/kg in PBS atipamezole subcutaneously in the back and were allowed to wake up in a cage with paper bedding, warmed by warm pads. For analgesia, 0.12 mg/kg buprenorphine was included in the Antisedan solution (injectable solution concentration: 0.225 mg/mL atipamezole, 0.015 mg/mL buprenorphine).

### Characterization of mice.

Characterization was performed on both eyes per mouse at 5 time points with 1-month intervals starting at the age of 1 month to 5 months of age using slit lamp photography, IVCM imaging of corneal layers, and OCT. At every time point, after the characterization of all eyes, 2 Het mice and 2 WT mice were taken out of their groups and sacrificed for tissue extraction, while all other animals were kept in their housing until reaching the next age time point. Obtained tissues were dissected, processed, or preserved according to the specific assay for which they were individually allocated. The specific assays and the corresponding tissue handling after extraction are described below in their respective sections. A clinical grading scale for AAK was applied to all Het mice, as previously reported (7). Briefly, grade 0 AAK is a complete and undamaged limbal border indistinguishable from a healthy corneal limbus, grade 1 AAK is where blood vessels cross the limbus while remaining within 1 mm of the limbal area, grade 2 AAK is where blood vessels invade the peripheral and paracentral cornea but preserve the central 2 mm of the cornea, grade 3 AAK is characterized by vascularization affecting the central cornea, and grade 4 is an advanced end-stage AAK with vascularization of the entire cornea and transformation of the anterior cornea into an opaque, thick, white, vascularized, irregular structure.

### Slit lamp photography.

Mouse eyes were examined using a rodent slit lamp camera (Micron III, Phoenix Technologies) for visualization and scoring of corneal haze and opacity. After initial slit lamp photography, 5 mg/mL tropicamide drops were applied for 4 minutes to dilate the iris, and the eyes were rephotographed after rinsing of the tropicamide drops with PBS eye drops. Corneal haze grading was assessed by slit lamp examination at each time point using a previously reported grading scale (33).

### IVCM imaging of corneal layers.

IVCM (Heidelberg retinal tomograph 3 with rostock corneal module, Heidelberg Engineering) was used to image the corneal layers to detect possible lesions, abnormal features, and inflammatory cell infiltrates while visualizing the corneal nerve plexus. After application of Viscotears Gel (2 mg/g polyacrylic acid), the laser head was brought in close contact with the cornea epithelium, and images were obtained and recorded throughout the corneal layers.

### OCT.

The Optovue iVue 100 OCT system with a corneal adaptor module was used on both eyes per mouse using the iVue system in the corneal pachymetry mode. The Fourier-domain OCT device operates at a scanning speed of 26,000 A-scans per second with a frame rate of 256 to 4,096 A-scans per frame. The axial and transverse resolutions of the iVue 100 OCT device are 5 μm and 15 μm, respectively (manual).

### Cornea tissue sample processing.

Upon sacrifice, eye bulbs were extracted with forceps. For histology, the whole eye was rinsed in PBS and immersed in 4% paraformaldehyde (PFA) in PBS at 4°C 2 hours, rinsed thoroughly with PBS, and kept in ethanol 70%. After being embedded in paraffin blocks, eyes were sectioned at 5 μm thickness, mounted to poly-l-lysine–coated microscopy slides, dried overnight at room temperature (RT), and further processed for histological-immunohistological-IF staining. In all other cases, the cornea was separated immediately and washed with ice-cold PBS. For protein analysis, the cornea was immersed in 80 μL RIPA buffer (Thermo Fisher Scientific 89901) supplemented with Halt protease and phosphatase inhibitors with 5 mM EDTA (Thermo Fisher Scientific 78442), then snap-frozen and stored at –80°C. For whole-mount staining, the corneas, after being immersed in 1.3% PFA at 4°C for 1 hour, were kept in PBS until processing for staining.

### Immunostaining and confocal fluorescence microscopy.

Sections from Het mouse corneas, including the attachment, were excluded from quantitative analyses to minimize the probable effect of attachment on the corneal phenotype outside the attachment zone. A standard protocol for section rehydration, antigen retrieval, blocking, and antibody staining was used. Briefly, antigen retrieval was performed in the DAKO PT LINK 200 machine; nonspecific epitope blocking was performed by 1% BSA (Sigma-Aldrich, Merck, A7906), 5% goat serum (Cell Signaling Technology, BioNordika, CST-5425S), 0.1% Tween 20 in PBS; primary Ab was incubated overnight at 4°C; secondary Abs were applied for 2 hours at RT; and SlowFade Diamond Antifade mountant with DAPI (Thermo Fisher Scientific 15451244) was used to mount immunostained slides or Eukitt quick-hardening mounting medium (Sigma-Aldrich, Merck, 03989) for IHC-stained slides. We specifically examined ΔNp63 and GPHA2 as indicators for LSCs, in addition to KRT12 as a marker for differentiated epithelial cells (negative marker for stem cells). For completeness, images were taken of the central cornea, including and excluding regions of keratolenticular adhesions in Het mice. For whole mounts, corneas were cut into 4 leaflets and then proceeded to IF protocol. Briefly, after nonspecific epitopes being blocked for 1 hour, the corneas were incubated in primary Ab overnight. Fluorophore-conjugated secondary Abs were applied for 2 nights. An LSM 800 microscope (Carl Zeiss AG) was used for imaging. Abs used are listed in Supplemental Table 2.

### Western blot.

For protein extraction, samples were homogenized using the TissueLyser LT bead mill system (QIAGEN) at 50 Hz shaking for 5 minutes, followed by sonication in ice-cold water for 10 minutes and a 20-minute centrifugation step (12,000*g*). Protein concentration was measured using the Pierce BCA Protein Assay kit (Thermo Fisher Scientific) following the instructions for a 10 μL sample volume. BIS-PAGE was performed using 4%–15% gradient precast gel (Bio-Rad 4561084 Mini-PROTEAN TGX). A total of 15 μg total protein per sample was loaded and mixed with 4× Laemmli buffer (Bio-Rad 1610747), 0.5 μL β-mercaptoethanol, and double-distilled H_2_O. Proteins were transferred in a PVDF membrane using Trans-Blot Turbo Mini 0.2 μm PVDF Transfer Packs (Bio-Rad 1704156), and standard membrane immunostaining procedures were followed, with primary and secondary antibody dilutions detailed in Supplemental Table 2. Bands were visualized in an ImageQuant LAS 500 Chemiluminescent Imaging System (GE Healthcare, now Cytiva), after being covered by 1 mL of Pierce ECL Western Blotting Substrate (Thermo Fisher Scientific 32106). Band densitometry on the photographed membranes was performed using the Image Lab v.6.1 software (Bio-Rad).

### Statistics.

ImageJ software (version 1.54, FIJI distribution) was utilized for all morphometric measurements of various aspects of microscopy images (34). Measurements in OCT images were performed within the OCT built-in software (v 3.2.1.8). Prism v.8.3.0 (GraphPad Software) was used for the statistical evaluation in group comparisons and to produce charts. Multiple-group comparisons were performed using 1-way ANOVA followed by post hoc Tukey’s multiple-comparison tests. Comparisons across 2 groups were performed using a 2-tailed unpaired *t* test. Results were reported as mean ± SEM. *P* value of 0.05 or less was considered significant. For statistical comparisons for the frequency of phenotypic findings between sexes, IBM SPSS version 29.0.2.0 software was used with the χ^2^ method with continuity correction and Bonferroni’s adjustment for multiple testing.

### Study approval.

Animal procedures were approved by the Linköping Regional Animal Ethics review board, Linköping, Sweden (Application Nos. 10940-2021, 06826-2024).

### Data availability.

All relevant data along with raw measurements of all parameters have been provided in the manuscript or in the supplemental information (see Supporting Data Values Excel file). All measurements are presented individually for each experiment sample. Libraries of microscopy photos are available from the corresponding author upon reasonable request with no restrictions on data availability.

## Author contributions

DJ performed experiments, data acquisition, tissue processing, and interpretation and drafted the manuscript. PM performed experiments, data and tissue processing, interpretation, image preparation, and partial writing and review of the manuscript. MA performed tissue processing and analysis and review of the manuscript. AD performed imaging and assisted with image processing, tissue processing, and review of the manuscript. YR performed image analysis and review of the manuscript. NL performed conceptualization, study design, design and coordination of experiments, funding acquisition, and data interpretation and assisted with manuscript writing and critical manuscript review and revisions.

## Supplementary Material

Supplemental data

Unedited blot and gel images

Supporting data values

## Figures and Tables

**Table 1:**
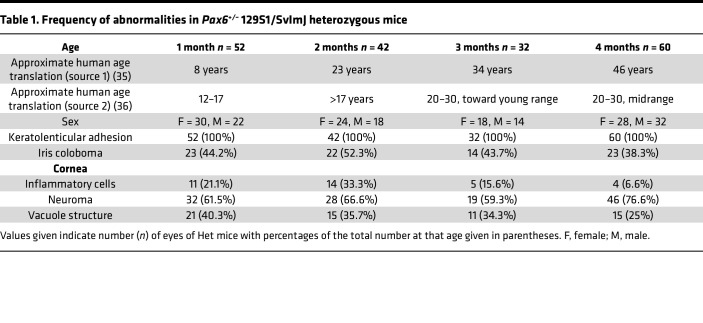
Frequency of abnormalities in *Pax6^+/–^* 129S1/SvImJ heterozygous mice

## Version 1 12/03/2024

In-Press Preview

## Version 2 01/23/2025

Electronic publication
