# Supplementary material for: A human-like model of aniridia-associated keratopathy for mechanistic and therapeutic studies
Source: JCI Insight. 2024 Dec 3;10(2):e183965. doi: 10.1172/jci.insight.183965 (PMC11790027; doi:10.1172/jci.insight.183965)
Supplement: Supplemental data [file jciinsight-10-183965-s010.pdf]

Supplemental Information for

A human-like model of aniridia-associated keratopathy for mechanistic and therapeutic studies

Dina Javidjam, Petros Moustardas, Mojdeh Abbasi, Ava Dashti, Yedizza Rautavaara, Neil Lagali*

Division of Ophthalmology, Department of Biomedical and Clinical Sciences, Linköping University, Linköping, Sweden

*Corresponding author: neil.lagali@liu.se

This supplemental information file contains the following Table and Figures:

Supplemental Table S1

Supplemental Table S2

Supplemental Figure S1

Supplemental Figure S2

Supplemental Figure S3

Supplemental Figure S4

Supplemental Figure S5

Frequency of abnormalities in Pax6 +/- 129S1/SvImJ heterozygous mice based on sex								
**Age**	1 month (n=52)		2 months (n=42)		3 months (n=32)		4 months (n=60)	
**Sex**	F	M	F	M	F	M	F	M
**Keratolenticular adhesion**	30 (100%)	22 (100%)	24 (100%)	18 (100%)	18 (100%)	14 (100%)	28 (100%)	32 (100%)
**Iris coloboma**	14 (47%)	9 (43%)	15 (63%)	7 (39%)	7 (39%)	7 (50%)	9 (32%)	14 (44%)
**Cornea**								
**Inflammatory cells**	7 (23%)	4 (19%)	4 (17%)	10 (56%)	2 (11%)	3 (21%)	2 (07%)	2 (06%)
**Neuroma**	18 (60%)	14 (67%)	17 (71%)	11 (61%)	10 (56%)	9 (64%)	20 (71%)	26 (81%)
**Vacuole structure**	14 (47%)	7 (33%)	8 (33%)	7 (39%)	7 (39%)	4 (29%)	8 (29%)	7 (22%)

Supplemental Table S1. Frequency of abnormalities in Pax6 +/- 129S1/SvImJ heterozygous mice according to sex. Data are presented as total number of mice of each sex with the indicated feature, and the corresponding percentage relative to the total number of mice with that sex in parentheses. No sex-dependent differences were noted for any parameter in Chi-Square tests ($P > 0.05$). F = female, M = male, n = number of mice at each given age.

Antibody	Host	Dilution	Supplier	Catalog No.
PAX6	Rabbit	1:100	Abcam	# ab195045
ΔNp63	Rabbit	1:50	Abcam	# ab203826
GPHA2	Mouse	1:50	Santa cruz	# sc-390194
KRT12	Mouse	1:100	Santa cruz	# sc-515882
β-III Tubulin	Mouse	1:200	Abcam	# ab78078
LYVE1	Rat	1:50	Invitrogen	# 14-0443-82
F4/80	Rat	1:150	Bio-Rad	#MCA497R
CD31	Rabbit	1:50	Abcam	# ab28364
COL4	Rabbit	1:50	Invitrogen	# PA1-28534
β-actin	Mouse	1:400	Thermo Fisher	# MA1-140
Ki-67	Rabbit	1:100	Abcam	#ab16667
MUC5AC	Mouse	1:50	Invitrogen	#MA5-12178
Anti-mouse IgG, Alexa Fluor 488 conjugated	Goat	1:400	Abcam	# ab150113
Anti-rabbit IgG, Alexa Fluor 647 conjugated	Goat	1:400	Abcam	# ab150079
Anti-rat IgG, Alexa Fluor 546 conjugated	Goat	1:400	Invitrogen	# A11081
Anti-Mouse IgG (H+L) poly-HRP conjugated	Goat	1:1000	Invitrogen	Pierce 32230

Supplemental Table S2. List of antibodies used in this study.

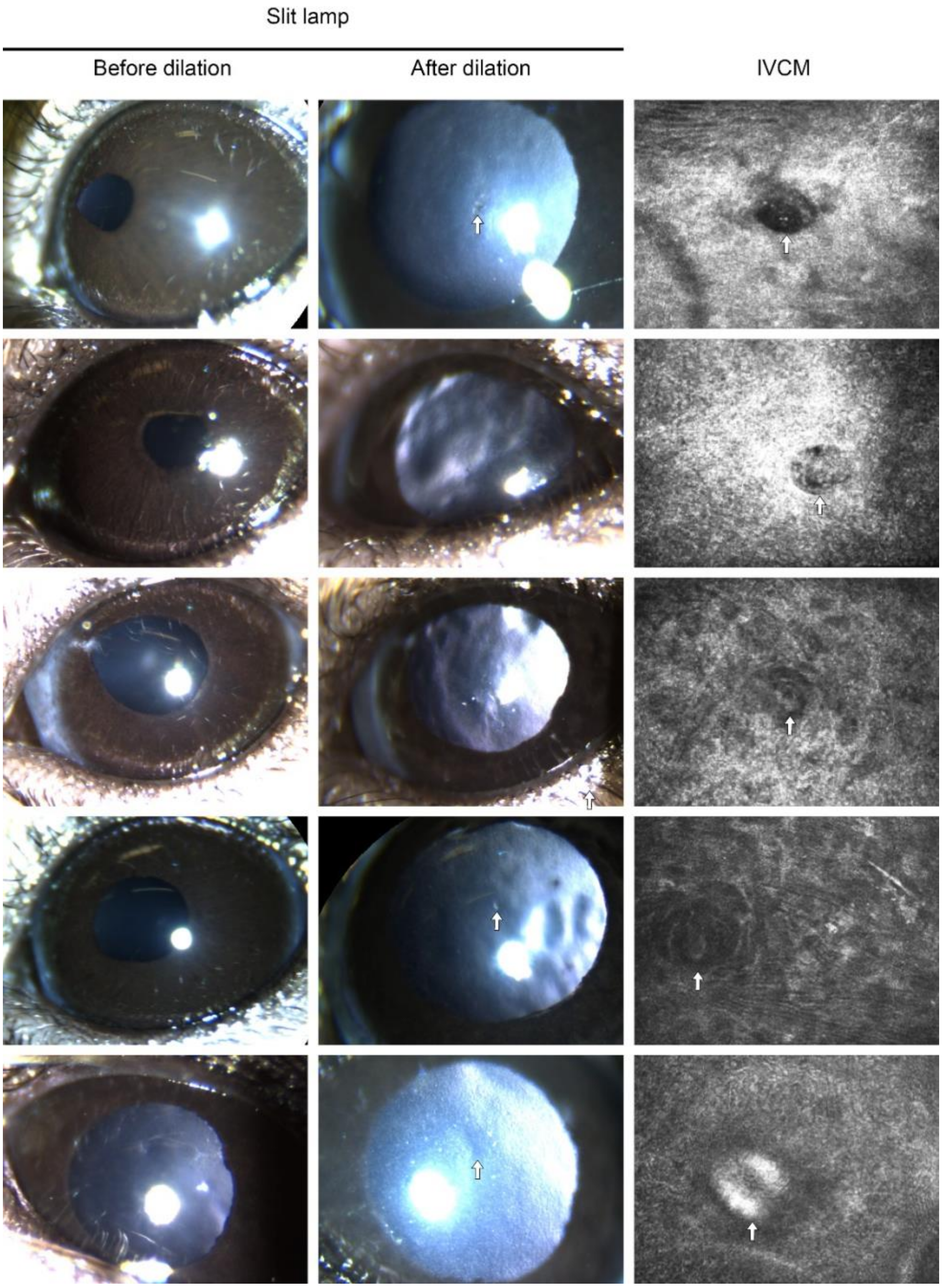

Supplemental Figure S1. Minor keratolenticular adhesions in different Het mice. The first two columns represent slit lamp images taken before and after dilatation with arrows indicating a small point of attachment. The final column shows the corresponding IVCM images with arrows indicating the posterior corneal stromal appearance of the point of attachment.

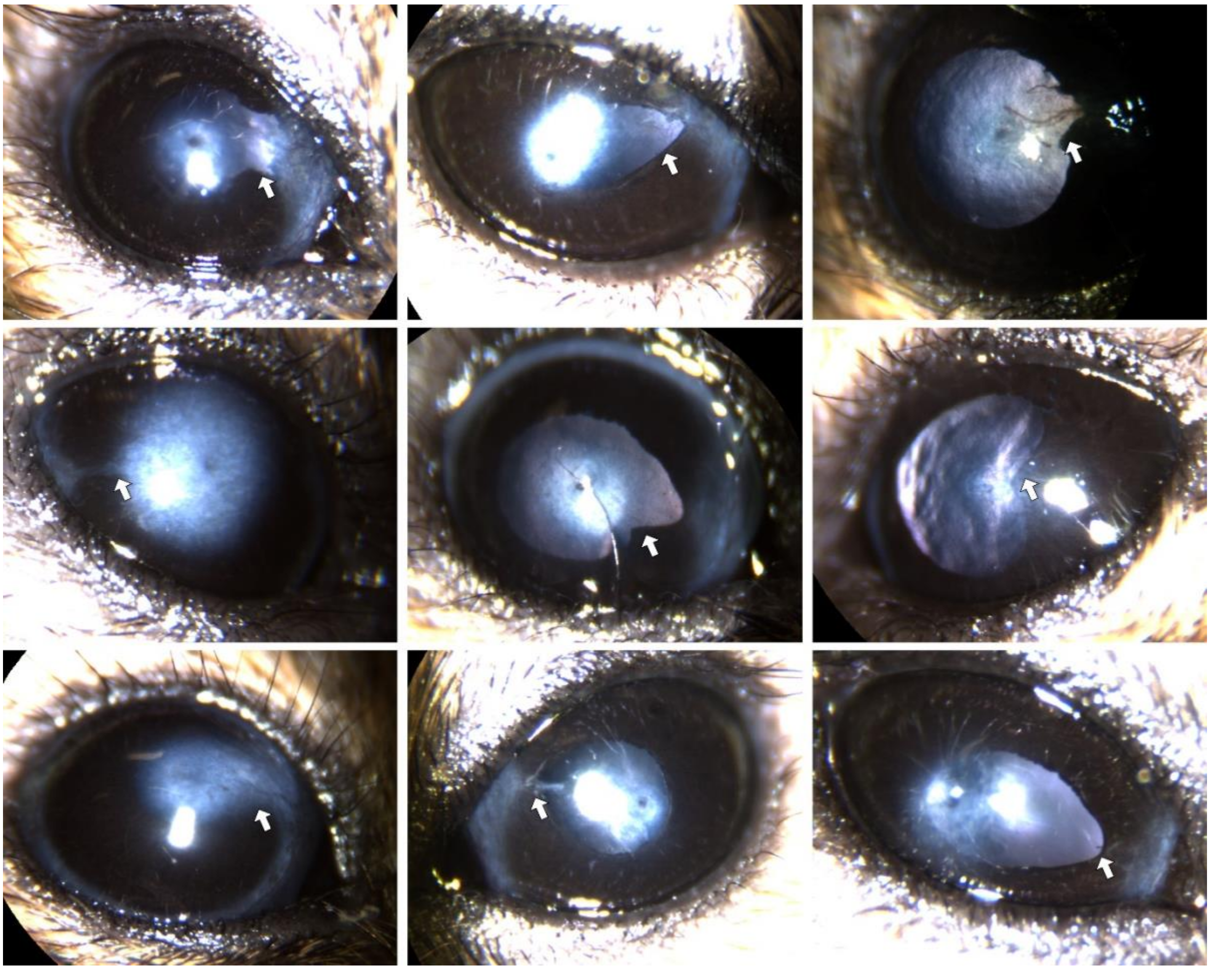

Supplemental Figure S2. Phenotypic spectrum of iris hypoplasia in different eyes of Het mice, with arrows indicating areas of iris anomalies.

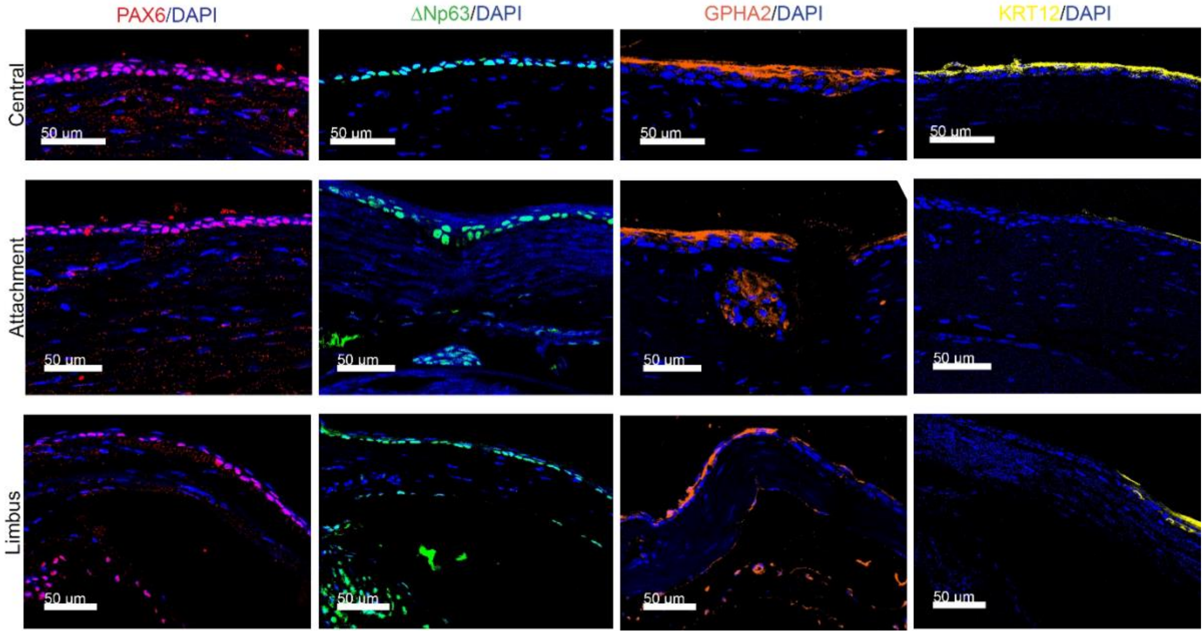

Supplemental Figure S3. Immunolocalization of PAX6, ΔNp63, GPHA2, and KRT12 in the corneal center, limbus, and central attachment zone in Wt and Het mice with Grades 3 and 4 AAK. (First column) PAX6. (Second column) ΔNp63. (Third column) GPHA2. (Forth column) KRT12. Nuclei are counterstained with DAPI (blue).

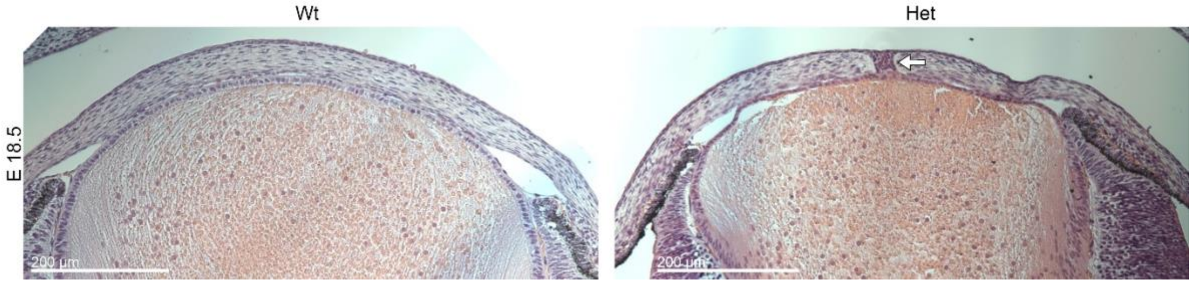

Supplemental Figure S4. H&E staining of E18.5 embryos from wild-type (Wt, left) and Het (right) mice. In the case of Het embryos, lens attachment is apparent at this late prenatal stage, with lens epithelial cells attached to and invading the corneal stroma (arrow), in contrast to the distinct separation of lens and cornea tissues in the Wt embryo. Note that in both Wt and Het sections, the collapsed central anterior chamber is an artifact of tissue processing.

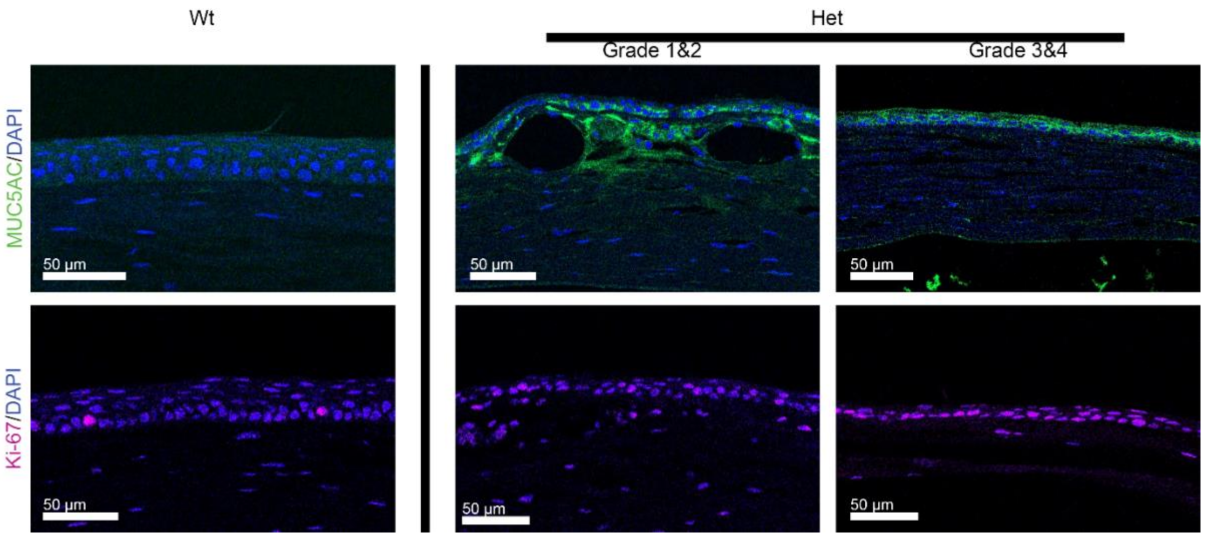

Supplemental Figure S5. Immunolocalization of conjunctival epithelial/goblet cell marker MUC5AC (top row) and proliferation marker Ki67 (bottom row) in Wt (First column) and Het mice with Grades 1 and 2 AAK (Second column) and Grades 3 and 4 (Third column). Nuclei are counterstained with DAPI (blue).
