# Supplementary figures and images for: A human-like model of aniridia-associated keratopathy for mechanistic and therapeutic studies
Source: JCI Insight. 2024 Dec 3;10(2):e183965. doi: 10.1172/jci.insight.183965 (PMC11790027; doi:10.1172/jci.insight.183965)

## Slide 1
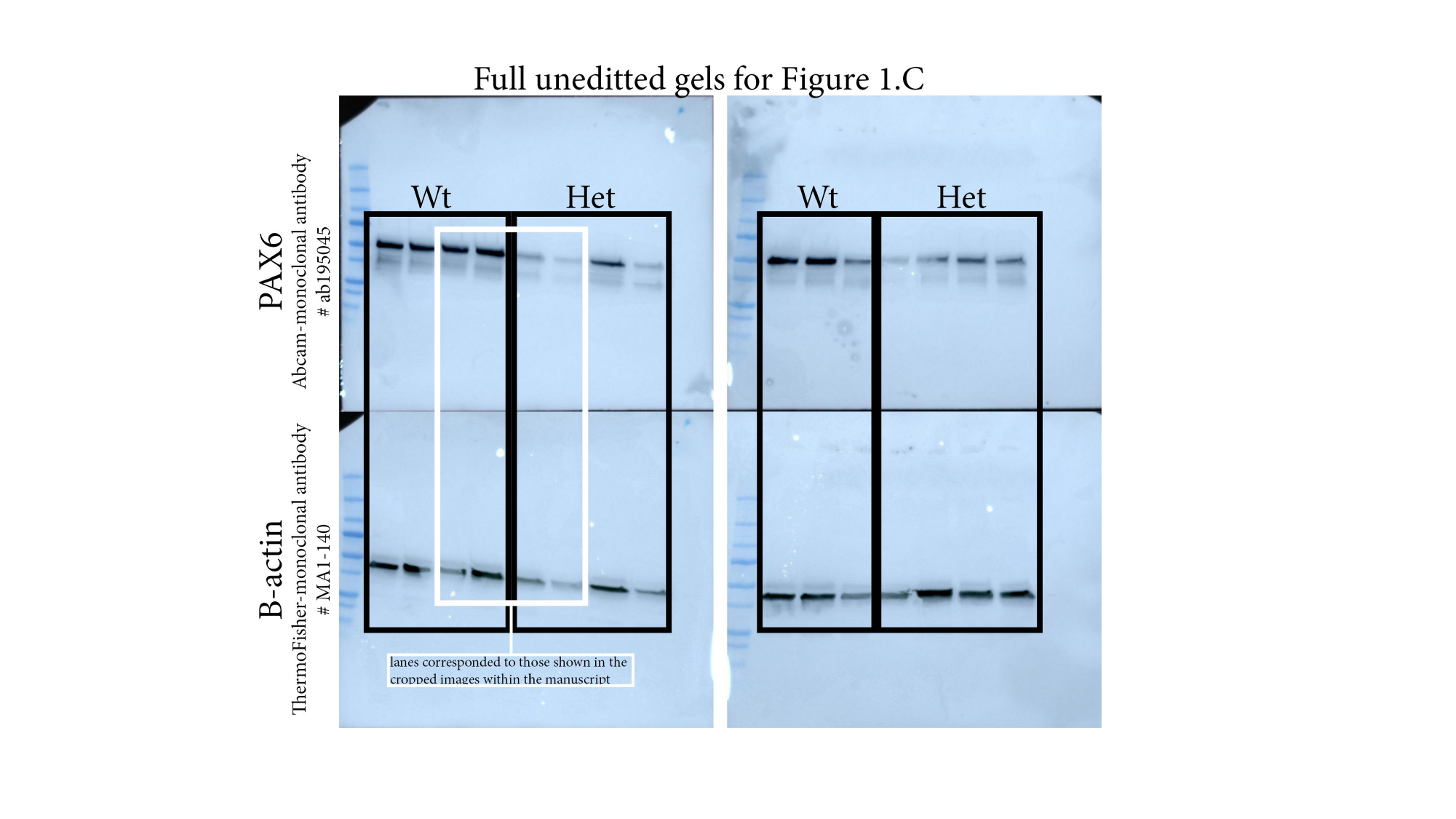

Supplement: Unedited blot and gel images [file jciinsight-10-183965-s011.pptx]
